# Identification of a plasma signature of psychotic disorder in children and adolescents from the Avon Longitudinal Study of Parents and Children (ALSPAC) cohort

**DOI:** 10.1038/tp.2017.211

**Published:** 2017-09-26

**Authors:** A O'Gorman, T Suvitaival, L Ahonen, M Cannon, S Zammit, G Lewis, H M Roche, I Mattila, T Hyotylainen, M Oresic, L Brennan, D R Cotter

**Affiliations:** 1 Department of Psychiatry, Royal College of Surgeons in Ireland (RCSI), Beaumont Hospital, Dublin, Ireland; 2 Institute of Food and Health, UCD School of Agriculture and Food Science, University College Dublin (UCD), Belfield, Dublin, Ireland; 3 Steno Diabetes Center, Gentofte, Denmark; 4 MRC Centre for Neuropsychiatric Genetics and Genomics, Cardiff University, Cardiff, UK; 5 Centre for Academic Mental Health, School of Social & Community Medicine, University of Bristol, Bristol, UK; 6 Division of Psychiatry, University College London, London, UK; 7 Nutrigenomics Research Group, UCD Conway Institute/UCD Institute of Food & Health, School of Public Health, Physiotherapy & Sports Science, University College Dublin (UCD), Belfield, Dublin, Ireland; 8 Department of Chemistry, Örebro University, Örebro, Sweden; 9 Turku Centre for Biotechnology, University of Turku and Åbo Akademi University, Turku, Finland; 10 School of Medical Sciences, Örebro University, Örebro, Sweden

## Abstract

The identification of an early biomarker of psychotic disorder is important as early treatment is associated with improved patient outcome. Metabolomic and lipidomic approaches in combination with multivariate statistical analysis were applied to identify plasma alterations in children (age 11) (38 cases vs 67 controls) and adolescents (age 18) (36 cases vs 117 controls) preceeding or coincident with the development of psychotic disorder (PD) at age 18 in the Avon Longitudinal Study of Parents and Children (ALSPAC). Overall, 179 lipids were identified at age 11, with 32 found to be significantly altered between the control and PD groups. Following correction for multiple comparisons, 8 of these lipids remained significant (lysophosphatidlycholines (LPCs) LPC(18:1), LPC(18:2), LPC(20:3); phosphatidlycholines (PCs) PC(32:2; PC(34:2), PC(36:4), PC(0-34-3) and sphingomyelin (SM) SM(d18:1/24:0)), all of which were elevated in the PD group. At age 18, 23 lipids were significantly different between the control and PD groups, although none remained significant following correction for multiple comparisons. In conclusion, the findings indicate that the lipidome is altered in the blood during childhood, long before the development of psychotic disorder. LPCs in particular are elevated in those who develop PD, indicating inflammatory abnormalities and altered phospholipid metabolism. These findings were not found at age 18, suggesting there may be ongoing alterations in the pathophysiological processes from prodrome to onset of PD.

## Introduction

Psychotic disorders (PD) are among the most severe and debilitating medical diseases, with schizophrenia being the most common, affecting ~0.5–1% of the global population.^1^ At present, the diagnosis of PD is subjective and there are no reliable biological diagnostic tests.^2^ Over the last decade, the psychosis field of research has shifted its focus to the prodrome or ‘at-risk mental state’ (‘ARMS’) in an attempt to identify and treat subjects at high risk of developing a psychotic illness. Investigations have indicated that 20–30% of these individuals will go on to develop schizophrenia over a 2–3 year period.^3^ Strong evidence supports early intervention as a means to alleviate and improve therapeutic outcome for individuals.^4, 5^ Owing to the multifactorial complexity of PD,^2, 6^ the identification of a disease signature and molecular biomarker(s) sensitive to the underlying pathogenic factors would be of great importance to assist in early detection and diagnosis^7^ but also to understand the mechanisms underlying the disease process, which has yet to be fully elucidated.^8^

Metabolomics, the global study of metabolites in cells, tissues or biofluids has emerged in recent years as a promising tool to identify abnormalities in psychotic illnesses. Metabolomic studies in schizophrenia and related psychoses have highlighted a number of metabolic perturbations such as glucoregulatory processes,^9, 10^ fatty acid and lipid metabolism,^11, 12, 13^ mitochondrial function,^14^ and proline^7^ and tryptophan metabolism.^15^ Although a comprehensive mapping of disturbances in metabolic pathways in PD is still a long way off,^16^ the most consistent findings involve pathways common to fatty acids and the pro-oxidant/antioxidant balance.^17^ A recent study by Rice and colleagues reported decreased levels of erythrocyte polyunsaturated fatty acid (PUFA) levels in young people at ultra-high risk of PD,^18^ providing additional evidence of the putative ω−3 PUFA deficiency syndrome. These studies have identified various metabolite signatures and have contributed to a developing and enhanced understanding of the disease mechanism. However, they generally focus on the adult population who have already transitioned to schizophrenia, with a majority being medicated, and so they are limited in terms of identifying early biomarker signatures of the disease.

The aim of this study was to apply metabolomic approaches to identify plasma alterations in children (age 11) and adolescents (age 18) in the ALSPAC cohort who developed PD at age 18. Such alterations may serve as potential early metabolic signatures/biomarkers.

## Materials and methods

### Study cohort

The study comprises individuals from the Avon Longitudinal Study of Parents and Children (ALSPAC) cohort. The ALSPAC cohort is a prospective general population cohort, and a rich resource of demographic, environmental and clinical data on the individuals involved.^19^ The initial cohort consisted of 14 062 births. Written informed consent was obtained prior to taking the plasma samples. Ethical approval for the study was obtained from the ALSPAC Ethics and Law Committee and the Local Royal College of Surgeons in Ireland (RCSI) Research Ethics Committees. Please note that the study website contains details of all the data that are available through a fully searchable data dictionary (http://www.bristol.ac.uk/alspac/researchers/access).

#### Measures of psychotic experiences and psychotic disorder

Psychotic experiences (PE) were identified at 11 and 18 years through the face-to-face, semi-structured Psychosis-Like Symptom (PLIKS) interview^20^ conducted by trained psychology graduates in assessment clinics, and were coded according to the definitions and rating rules for the Schedules for Clinical Assessment in Neuropsychiatry, Version 2.0 (Organisation 1994). Interviewers rated PEs as not present, suspected or definitely psychotic. Cases of psychotic disorder (PD) were defined as individuals with definite PEs that were not attributable to the effects of sleep or fever and when the PE occurred at least once per month over the past 6 months and caused severe distress, had a very negative effect on social/occupational function, or led to help seeking from a professional source.^20^ Control samples from age-matched individuals without suspected or definite PEs, or PD at age 18 were randomly selected to match cases (Table 1).

### Plasma availability in age 11 samples

We identified 38 individuals with PD at age 18 (cases) who had plasma samples at age 11 available for the metabolomic/lipidomic analysis. Control samples (*n*=67) from age and body mass index (BMI)-matched individuals without suspected or definite PEs at age 11, or PD at age 18 were randomly selected to match cases (Table 1).

### Plasma availability in age 18 samples

We identified 36 individuals with PD at age 18 (cases) who had plasma samples at age 18 available for the metabolomic/lipidomic analysis. Control samples (*n*=117) from age and BMI-matched individuals who did not have PD at age 18, or suspected or definite PEs at age 11 were randomly selected to match cases (Table 1). Of the PD cases, 25 individuals plasma samples were present at both age 11 and 18. Therefore, considering that there were 38 PD samples at age 11, and 36 PD samples at age 18, overall there were a total of 49 individual PD subjects studied.

### Plasma sample collection

Non-fasting (age 11) and fasting (age 18) blood samples were collected from the participants into heparin Sarstedt S-Monovette tubes (Sarstedt, Numbrecht, Germany), stored on ice for a maximum of 90 min until processed. Post centrifugation, the plasma samples were stored at −80 °C until further analyses.

### Global lipidomic analysis

The plasma samples were analysed for global profiling of lipids using a method developed specifically for lipidomics analyses. The samples were prepared following the previously published Folch procedure^21^ with minor modifications. Briefly, 10 μl of 0.9% NaCl, 100 μl of CHCl_3_:MeOH (2:1, v/v) and 20 μl of a 3.5 μg ml^−1^ working standard solution of chosen lipid standards (for quality control and data normalisation purposes) were added to 10 μl of each plasma sample. The standard solution contained the following compounds: 1,2-diheptadecanoyl-sn-glycero-3-phosphoethanolamine (PE(17:0/17:0)), *N*-heptadecanoyl-d-erythro-sphingosylphosphorylcholine (SM(d18:1/17:0)), *N*-heptadecanoyl-d-erythro-sphingosine (Cer(d18:1/17:0)), 1,2-diheptadecanoyl-sn-glycero-3-phosphocholine (PC(17:0/17:0)), 1-heptadecanoyl-2-hydroxy-sn-glycero-3-phosphocholine (LPC(17:0)) and 1-palmitoyl-d31-2-oleoyl-sn-glycero-3-phosphocholine (PC(16:0/d31/18:1)). These were purchased from Avanti Polar Lipids (Alabaster, AL, USA). In addition, 1,2-dimyristoyl-sn-glycero-3-phospho(choline-d_13_) (PC(14:0/d13)) was purchased from Sigma Aldrich (Wicklow, Ireland) and Tripalmitin-1,1,1-13C3 (TG(16:0/16:0/16:0)-13C3) and Trioctanoin-1,1,1−13C3 (TG(8:0/8:0/8:0)−13C3) from Larodan (Solna, Sweden) for the same purpose. The samples were vortex mixed and allowed to stand on ice for 30 min after which they were centrifuged (9400 *g*, 3 min, 4 °C). Overall, 60 μl from the lower layer of each sample was then transferred to a glass vial with an insert and 60 μl of CHCl_3_:MeOH (2:1, v/v) was added. The samples were then stored at −80 °C until analysis.

Calibration curves (at concentration levels of 100, 500, 1000, 1500, 2000 and 2500 ng ml^−1^) for quantification of lipids were prepared using 1-hexadecyl-2-(9Z-octadecenoyl)-sn-glycero-3-phosphocholine (PC(16:0e/18:1(9Z))), 1-(1Z-octadecenyl)-2-(9Z-octadecenoyl)-sn-glycero-3-phosphocholine (PC(18:0p/18:1(9Z))), 1-octadecanoyl-sn-glycero-3-phosphocholine (LPC(18:0)), 1-(1Z-octadecenyl)-2-docosahexaenoyl-sn-glycero-3-phosphocholine (PC(18:0p/22:6)), 1-stearoyl-2-arachidonoyl-sn-glycero-3-phosphoinositol (PI(18:0/20:4)) and 1-stearoyl-2-linoleoyl-sn-glycerol (DG(18:0/20:4)) from Avanti Polar Lipids, 1-Palmitoyl-2-Hydroxy-sn-Glycero-3-Phosphatidylcholine (LPC(16:0)) from Larodan, and 1,2,3-Triheptadecanoylglycerol (TG(17:0/17:0/17:0)) and 3β-hydroxy-5-cholestene 3-linoleate (ChoE(18:2)) from Sigma Aldrich.

The samples were analysed using an ultra-high-performance liquid chromatography quadrupole time-of-flight mass spectrometry method (UHPLC-Q-TOF-MS). The UHPLC system was a 1290 Infinity system from Agilent Technologies (Santa Clara, CA, USA), which was equipped with a multisampler (maintained at 10 °C) using 10% DCM in MeOH and ACN:MeOH:IPA:H_2_O (1:1:1:1, v/v/v/v)+0.1% HCOOH as needle wash solutions after each injection for 7.5 s each, a quaternary solvent manager and a column thermostat (maintained at 50 °C). Separations were performed on an ACQUITY UPLC BEH C18 column (2.1 mm × 100 mm, particle size 1.7 μm) by Waters (Milford, CT, USA). The flow rate was 0.4 ml min^−1^ and the injection volume was 1 μl. H_2_O+1% NH_4_Ac (1M)+0.1% HCOOH (A) and ACN:IPA (1:1, v/v)+1% NH_4_Ac+0.1% HCOOH (B) were used as the mobile phases for the gradient elution. The gradient was as follows: from 0 to 2 min 35–80% B, from 2 to 7 min 80–100% B and from 7 to 14 min 100% B. Each run was followed by a 7 min re-equilibration period under initial conditions (35% B). The mass spectrometer was a 6550 iFunnel quadrupole time-of-flight (Q-TOF) from Agilent Technologies (Agilent) interfaced with a dual jet stream electrospray (dual ESI) ion source. Nitrogen generated by a nitrogen generator (PEAK Scientific, Renfrewshire, Scotland, UK) was used as the nebulising gas at a pressure of 21 psi, as the drying gas at a flow rate of 14l min^−1^ (at 193 °C) and as the sheath gas at a flow rate of 11l min^−1^ (at 379 °C). Pure nitrogen (6.0) from Praxair (Fredericia, Denmark) was used as the collision gas. The capillary voltage and the nozzle voltage were kept at 3643 and 1500 V, respectively. The reference mass solution including ions at *m/z* 121.0509 and 922.0098 was prepared according to instructions by Agilent and it was introduced to the mass spectrometer through the other nebuliser in the dual ESI ion source using a separate Agilent series 1290 isocratic pump at a constant flow rate of 4 ml min^−1^ (split to 1:100 before the nebuliser). The acquisition mass range was *m/z* 100–1700 and the instrument was run using the extended dynamic range with an approximate resolution of 30 000 FWHM measured at *m/z* 1521.9715 (which is included in the tune mixture) during calibration of the instrument. MassHunters B.06.01 (Agilent) software was used for all data acquisition.

Quality control was performed throughout the dataset by including blanks, pure standard samples, extracted standard samples and control plasma samples. Relative standard deviations (%RSDs) for retention times and peak areas for lipid standards representing each lipid class in the plasma samples and in the pure standard samples were calculated. The %RSDs for the retention times were on average 0.1% for both the plasma samples and for the pure standards samples. The %RSDs for the peak areas were within accepted analytical limits at averages of 17.5 and 16.6% for the plasma samples and for the pure standard samples, respectively. This shows that the method is reliable and repeatable throughout the sample set.

Lipidomic data were pre-processed with MZmine 2 and peaks were identified based on the internal peak library. Peak areas were normalised by lipid class-specific internal standards and quantified with R based on the inverse-weighted linear model. Internal standard peaks were detected in a targeted way from the standards runs. The internal MS library’s retention times were corrected with R to match the study with a linear correction based on the observed retention times in the standards runs. Other peaks in the sample runs were processed in a non-targeted way as: import of the mzML files, mass detection, chromatogram builder, chromatogram deconvolution, isotopic peak grouper, peak filter, peak list row filter, join aligner, peak list row filter, gap filling, peak list filter, identification with custom data base search. The peak table was filtered in R, allowing the peak to be missing in each of the eight batches in a maximum 50% of the samples. Remaining missing values were imputed with feature-wise half-the-minimum. All lipid metabolites, that were present (non-zero value) in more than 75% of samples were included in the data analyses.

### Global metabolomic analysis

Polar metabolites were analysed in the plasma samples using comprehensive two-dimensional gas chromatography combined with time-of-flight mass spectrometry (GC × GC-TOFMS, a LECO Pegasus 4D equipped with a cryogenic modulator from LECO, St. Joseph, MI, USA). Specifically, 400 μl methanol and 10 μl internal standard mixture heptadecanoic acid (175 mg l^−1^), dl-valine-d8 (36 mg l^−1^), succinic acid-d4 (59 mg l^−1^) and dl-glutamic acid-d5 (110 mg l^−1^) were added to 30 μl of plasma samples. The samples were vortex mixed and centrifuged for 5 min at 10 000 r.p.m. and half of the supernatant was evaporated to dryness. This was followed by two step derivatisation using methoximation and silylation. Specifically, the samples were derivatised by adding first 25 μl methoxamine MOX (45 °C, 60 min) and then 25 μl *N*-trimethylsilyl-*N*-methyl trifluoroacetamide MSTFA (45 °C, 60 min). Finally, 50 μl of a retention index standard mixture (*n*-alkanes, *c*=8 mg l^−1^) with an injection standard (4,4′-dibromooctafluorobiphenyl, *c*=10 mg l^−1^), both in hexane, was added to the mixture. The calibration consisted of six points for each quantified metabolite.

The columns were as follows: a methyl deactivated retention gap (1.5 m × 0.53 mm i.d.) was connected to 10 m × 0.18 mm Rtx-5 (i.d. diameter 0.18 mm, df_phase_ thickness 0.20 μm) and to 1.5 m × 0.1 mm BPX-50 (i.d. 0.1 mm, df_phase_ thickness 0.1 μm). Helium was used as the carrier gas at a constant pressure mode (40psig). A 4 s separation time was used in the second dimension. The temperature programme was as follows for the first-dimension column: 50 °C (2 min), at 7 °C min^−1^ to 240 °C and at 25 °C min^−1^ to 300 °C (3 min). The second-dimension column temperature was 15 °C higher than the corresponding first-dimension column throughout the programme.

The analytical method used allows for combined targeted and untargeted analysis, where a selected subset of metabolites can be quantified. In the present study, quantitation of 23 target metabolites (stearic acid, oleic acid, linoleic acid, palmitic acid, citric acid, glutamic acid, 3,4-dihydroxybutanoic acid, 3-hydroxybutyric acid, 2,4-dihydroxybutanoic acid, threonine, phenylalanine, serine, lactic acid, methionine, glycine, isoleucine, leucine, valine, proline, 2-hydroxybutyric acid, cholesterol, arachidonic acid and alanine) was done by external calibration curves for each individual metabolite.

ChromaTOF vendor software (LECO) was used for within-sample data processing, and the Guineu software (https://code.google.com/p/guineu/) was used for alignment, normalisation and peak matching across samples.^22^ The normalisation was performed by correction for internal standards and specific target metabolites were additionally quantified using external calibration curves. Other mass spectra from the GC × GC-TOFMS analysis were searched against the NIST 14 Mass Spectral Library and Golm Metabolome Database,^23^ using also retention index data in the identification. Artefact peaks due to chemical background and compounds outside the linear range of the method were removed from the dataset. Control serum samples (*n*=32 for human) and pure standards (to monitor the instrument performance and robustness) were analysed together with the samples. The relative standard deviation (RSD) for internal standards, spiked into the samples, was on average of 15.7% for the plasma samples. The RSD% of the quantified metabolites in the control serum samples (*n*=32) was on average 23.4%. The RSD% was 13.3% for isoleucine, 11.2% for leucine and 24.2% for valine in control serum samples (*n*=4), and for internal standards in mice serum the RSD was on average 16.6%. Neither sample preparation nor analysis order showed any significant effect on the results.

All plasma metabolite peaks that were present (non-zero value) in more than 75% of samples were included in the data analyses, including the unidentified ones. The unidentified peaks were annotated with their structural class from the Golm Metabolome Database using functional group prediction based on the fragmentation patterns.^22, 23^

### Targeted metabolomic analysis

A targeted metabolomic approach was taken to target a specific metabolic pathway, that is, the tricarboxylic acid (TCA) cycle (citric acid, succinic acid, fumaric acid, malic acid, α-ketoglutaric acid). A subset of control samples at both ages 11 and 18 were used for the analysis. This resulted in the following study numbers: PD at age 11 (38 controls vs 38 cases) and PD at age 18 (36 controls vs 36 cases) (Supplementary Table 4). Briefly, 50 μl of 0.1 mg ml^−1^ of Labelled internal standards (succininc acid-2,2,3,3-d_4_, fumaric acid-2,3-d_2_, adipic acid-2,2,5,5-d_4_ and dl-malic acid-2,3,3-d_3_, Sigma Aldrich) were added to 100 μl of plasma. To this, 440 μl of anhydrous EtOH, 100 μl pyridine, 500 μl of deionised H_2_O and 150 μl of ethylchloroformate (ECF) was added and samples were vortexed. To this, 1000 μl of CHCl_3_ was added. The pH was then adjusted to 5–6 by adding 7m NaOH solution and shaken for 5 s. Overall, 100 μl of ECF was added and vortexed for 20 s. To this, 2 ml of deionised H_2_O was added and vortexed. The samples were then centrifuged at 1700 *g* for 3 min at 4 °C. The CHCl_3_ layer was transferred to a 10 ml glass tube containing anhydrous sodium sulphate drying reagent. Following drying, samples were analysed using an Agilent 7200 Q-TOF GC/MS system (Agilent, Santa Clara, CA, USA).

Calibration was achieved by comparison of metabolite peak areas with reference to an external standard (TCA cycle metabolite library, Sigma Aldrich) and by comparison of their mass spectra with those in the NIST library (version 2.0). For quality control purposes, an aliquot from a pool of plasma was extracted and analysed in parallel with each batch of samples. Quantification of the TCA metabolites was carried out using the Masshunter TOF quantitative analysis software (version B.07.01, Agilent).

### Statistical analyses

Censored regression analysis (SAS, version 9.3, Cary, NC, USA) was applied to the datasets to identify significant differences (*P*<0.05) in lipid and metabolite levels between the control and PD groups at both age groups, adjusting for gender and BMI. Significant lipids and metabolites were corrected for multiple comparisons using the Benjamini–Hochberg step-up procedure in R (version 3.2.2) (*P*<0.1). Censored regression analysis was applied as the majority of lipids and metabolite levels were not normal-distributed and were reported in varying concentration ranges among participants. Individual lipid levels were visualised using the beanplot algorithm implemented in R. A beanplot gives information on the mean lipid level within each group, the density of the data-point distribution and illustrates individual data points.^11^ Cluster analysis was performed using the Mclust package^24^ in R (version 3.2.2), which uses Gaussian mixture modelling fitted via the expectation-maximisation (EM) algorithm for model-based clustering.^24^ Prior to clustering, the data were normalised using a logarithmic transformation.

For the targeted metabolomic analysis, metabolite levels between groups were compared using general linear models gender and BMI as covariates (SPSS version 20, IBM, Armonk, NY, USA).

The levels of the lipids in the PD case group were analysed relative to the control group as a function of the acyl chain length and double bond content within each lipid group. The level ratio was computed by first computing each lipid’s average level within each subject group, and the computing the ratio of these average levels between the PD case and the control groups. Each lipid’s level ratio was shown on the y-axis of two lipid group-specific figures for each time point: one with the number of carbon atoms in the lipid’s acyl chain on the *x* axis and another with the number of double bonds in the lipids’ acyl chain on the *x* axis. These figures were used for the qualitative evaluation of changes in the lipids that could be related to the chain length and level of saturation.

## Results

The final lipidomic datasets used for analysis included a total of 179 lipids from the following lipid classes: cholesterol esters (CEs), lysophosphatidlycholines (LPCs), phosphatidlycholines (PCs), phosphatidylethanolamines (PEs), sphingomyelins (SMs), triglycerides (TGs), a diacylglycerol (DG) and a phosphatidylinositol (PI).

### Age 11 and 18 lipidomic signatures of PD

**Figure 1:**
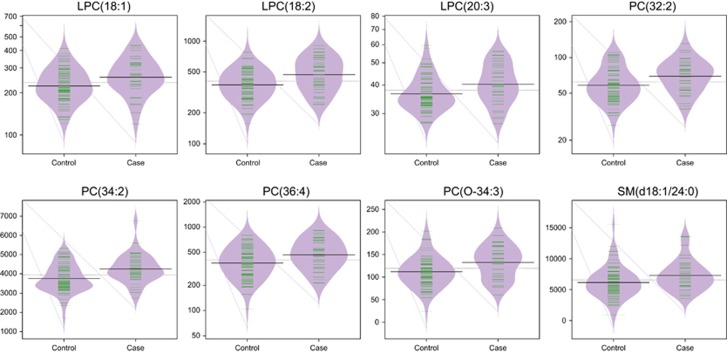
The levels of significant lipids at FDR level for age 11 samples.

**Figure 2:**
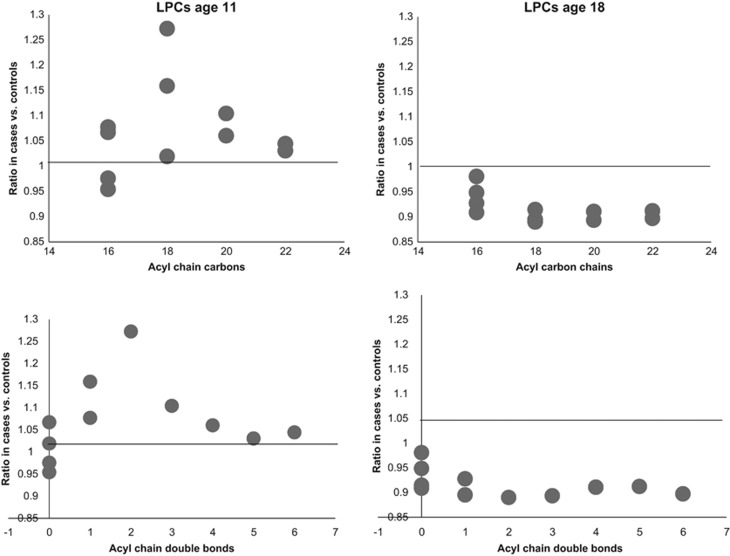
The relationship between psychotic disorder and acyl chain content in lipid species. The mean ratio of lipid levels in cases vs controls in plasma samples (ages 11 and 18) for lysophosphatidlycholines (LPCs).

**Figure 3:**
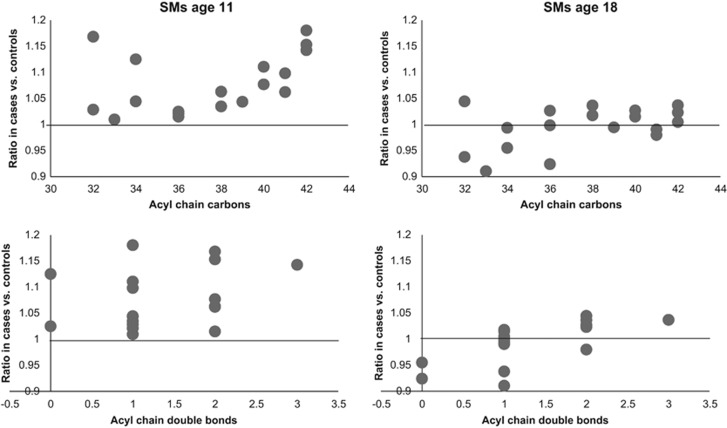
The relationship between psychotic disorder and acyl chain content in sphingomyelins (SMs). The mean ratio of lipid levels in cases vs controls in plasma samples (ages 11 and 18) for SMs.

At age 11, a total of 32 lipids were significantly altered between controls and cases (nominal *P*-value <0.05). These lipids included three CEs, five LPCs, 19 PCs, four SM and a TG (Table 2). All of these lipids, with the exception of TG(56:7), were elevated in the PD group. Eight of these passed FDR significance (*P*-value <0.1) (Figure 1). Relationships with the acyl chain length of lipid species was examined (Figures 2 and 3; Supplementary Figure 1). LPCs of lower carbon numbers and double bond content were higher in the PD group. An upward sloping relationship was seen in the mean ratio of SM levels in cases vs controls for higher carbon numbers. Clear patterns are not seen for the PCs and TGs, although the figure does highlight that the PCs are elevated in the PD group and TGs are downregulated, particularly so for those with a lower number of carbon double bonds.

The global lipidome was then evaluated by clustering the data into a set of lipid clusters. Cluster analysis identified seven lipid clusters (LC) and a description of each cluster is given in Table 3. Three of the identified clusters were significantly higher in the PD case group. LC4 was dominated by LPCs (*P*-value=0.025), LC6 was dominated by both PCs and CEs (*P*-value=0.036), whereas LC7 was a cluster containing PCs (*P*-value=0.006).

At age 18, a total of 23 lipids were significantly different between the control and PD groups (Table 4; nominal *P*-value <0.05). These lipids were three LPCs, thirteen PCs, one PE, three SMs and three TGs. All of these lipids, with the exception of PC(36:4), were decreased in the PD group (Figure 2), although no clear pattern with respect to the acyl carbon chain length or double bond content was observed. The lipid cluster LC4 was significantly decreased in the PD group (*P*-value=0.017) and was dominated by LPCs (Supplementary Table 1). None of these however passed significance at the selected FDR threshold of 0.1.

### Age 11 and 18 polar metabolomic signatures of PD

The metabolomic datasets used for analysis contained a total of 151 metabolites. At the age of 11, a total of 8 metabolites were significantly different between the control and PD groups, 6 of which were significantly decreased (1-monopalmitin, 2,3-dihydroxybutanoic acid, d-(−)-ribofuranose, ethanolamine, hydroxylamine and ribitol) and two significantly elevated (sugar derivatives) in the PD group (Table 5), although none were significant at the selected FDR threshold. Cluster analysis identified six metabolite clusters (CL), none of which were significant between study groups (Supplementary Table 2).

At the age of 18, a total of 19 metabolites were significantly different between the case and control groups, none at FDR level (Table 6). These metabolites mainly belonged to the following classes of metabolites; organic acids, fatty acids and sugar/sugar derivatives and were all significantly decreased in the PD group with the exception of glycine and levoglucosan which were elevated in the PD group. 1-Monopalmitin is the only common significant metabolite between the studies and was found to be decreased in PD groups at both ages. The metabolite cluster CL1 was significantly decreased in the PD group (*P*-value=0.034) and contained 22 metabolites consisting of fatty acids, organic acids and unknown metabolites (Supplementary Table 3).

Targeted analysis of the TCA cycle metabolites revealed no significant difference between the study groups at the ages of 11 and 18. At the age of 18, succinic acid was significantly decreased in the PD group (*P*-value=0.04) (Table 7).

## Discussion

Our findings indicate that the lipidome is altered between subjects with PD compared to subjects who do not experience any PEs, particularly at the age of 11, giving promise to the potential of an early biomarker signature of PD. At the age of 11, a number of LPCs were found to be significantly elevated in the PD group. This is indicative of altered phospholipid metabolism, which has been considered as the pathophysiological basis of schizophrenia for some time.^13^ A number of studies have reported impaired phospholipid levels in schizophrenia subjects, although the specific results have not been consistent.

LPC, an inflammatory phospholipid generated by hydrolysis of PC via the actions of phospholipase A_2_ (PLA_2_), promotes inflammatory effects such as expression of endothelial cell adhesion molecules, growth factors, chemotaxis and activation of monocytes/macrophages.^25^ LPC has been suggested to have a functional role in the pathogenesis of various diseases including atherosclerosis,^26^ diabetes^27^ and systematic autoimmune diseases.^28^ The association between inflammatory abnormalities and PD has been found repeatedly in many studies, which reported increased levels of pro-inflammatory compounds (mainly cytokines) in the serum, plasma and cerebrospinal fluid of patients with psychosis^29^ or who transition to schizophrenia from the at-risk mental state.^30^ In support of the inflammatory theory in relation to psychotic outcome in the ALSPAC cohort, Khandaker *et al.*^31^ reported higher levels of the systematic inflammatory cytokine marker IL-6 in childhood, which was associated with an increased risk of developing psychosis in young adulthood.

Lipidomic studies have previously identified elevated plasma levels of LPCs (16:0, 18:0, 18:1 and 18:2) in first-episode neuroleptic naive schizophrenia patients as compared to healthy controls.^12^ Of the reported significant LPCs, two were also significantly elevated in this study at the age of 11 (LPC 18:1 and LPC 18:2), which suggests that LPCs with lower carbon numbers and fewer double bonds are associated with risk of PD. An earlier study reported increased platelet membrane LPC in schizophrenia patients.^32^ However, there are inconsistencies in studies, with one study reporting diminished levels of LPCs in the serum of schizophrenia patients compared to their co-twins as well as healthy controls.^11^ Furthermore, there are some noteworthy difference between this study and previous ones, particularly relating to the age of the subjects and the diagnosis. The samples studied in the current investigation were donated from participants at the ages 11 and 18 and were thus very different in terms of medication exposure and duration of psychotic illness from those of first episode or chronic schizophrenia. In comparison, the mean age of first-episode schizophrenia patients in the study by Cai and colleagues was 27.5±9.5 years (*n*=11). In the twin study, which involved twin pairs discordant for schizophrenia, the participants had an average age of 51 years (*n*=19), with the majority taking antipsychotic medication. The differences between these studies could explain some of the inconsistencies in the reported findings related to the direction of the LPCs and also highlights the need for validation studies for biomarker confirmation. Identification of the biomarkers at a young age prior to conversion to schizophrenia would be of more benefit clinically to assist early detection and diagnosis.^7^

A total of four PCs were found to be significantly elevated in the plasma of the PD group at age 11. At age 18, the PCs were decreased in the PD group, although not significant at the selected FDR threshold. A few studies have reported PCs to be related to psychotic outcome, with most of the studies reporting reduced levels in schizophrenia.^33, 34^ The finding of elevated LPCs and PCs at age 11 and subsequent decreased levels relative to the controls (although not significant at FDR level) at age 18 suggest that biomarker profiles may change with age. Furthermore, it suggests that the pathophysiological process can alter over time and with proximity to disease onset.^35^ A similar finding was reported in type 1 diabetes where increased LPCs were identified in children who later progressed to the disease. Following seroconversion to autoantibody positivity, the metabolic profiles of the participants were partially normalised, indicating that early biomarkers may be useful in diabetes prevention strategies.^36^ It has been suggested elsewhere that maybe the ideal biomarkers are involved in aetiological pathways and are subsequently likely to change according to disease status.^37^

In terms of the metabolome, very few metabolite differences were identified between control and PD groups (ages 11 and 18), with none significant at FDR level. 1-monopalmitin was reported to be decreased in the PD group at both ages and is a lipid implicated in membrane integrity and stability and an energy storage source.^38^ Citric acid and succinic acid (targeted metabolomic approach) were found to be reduced in the PD group at age 18. A number of previous studies have reported citric/citrate acid levels to be reduced in serum and urine samples in schizophrenia patients, with one study finding elevated levels in serum in a schizophrenia cohort, indicating a compromised energy metabolism.^12, 16^ Decreased levels of citric acid and succinic acid indicate a dysfunction in TCA cycle activity and a deficit in mitochondrial respiration.^16^ A study comparing healthy controls vs schizophrenia pre-frontal cortex tissue reported that aerobic energy pathways were significantly decreased with reduced protein and transcript expression of TCA and oxidative phosphorylation enzymes,^14^ confirming compromised energy metabolism in individuals with schizophrenia.^39, 40, 41^

A major strength of this investigation is the ALSPAC cohort upon which it was based. The ALSPAC cohort includes detailed longitudinal clinical assessments and biosampling. The plasma samples were collected at age 11, prior to the development of psychotic disorder, and were not subject to confounds of drug exposure and chronic illness. Our observation of an altered lipidome at age 11, therefore, highlights a perturbed metabolism related to inflammation in the PD group, which may be an early biomarker signature of the disease, with the LPCs representing potential future early biomarkers of PD. The detected associations indicate that lipidomic profiles are useful for identifying important disease signatures. However, as a step to move these signatures closer to utilisation in the clinic validation in an external cohort is necessary. Notwithstanding this, the present study is an important step towards the identification of a signature associated with the development of PD.

## Figures and Tables

**Table 1:** An overview of the ALSPAC study population characteristics

	*Controls age 11* *(*n=*67)*	*Cases age 11* *(*n=*38)*	*Controls age 18* *(*n=*117)*	*Cases age 18* *(*n=*36)*
BMI±s.d.	17.73±2.53	18.2±3.36	22.69±3.51	23.12±3.74
Male:female	39M: 28F	8M:30F	58M:59F	8M:28F

Abbreviations: BMI, body mass index; s.d., standard deviation.

**Table 2:** Differential plasma lipids between control and PD groups at age 11

*Lipid name*	*Control (*n=*67)*	*Cases (*n=*38)*	P-*value*	*FDR-adjusted*
CE(18:2)	13481.42±4506.22	15773.88±3728.61	0.0392	0.242
CE(18:2)+unknown CE (667.6219)	6568.58±2222.39	7967.17±2181.29	0.0313	0.2391
LPC(16:1)	38.27±5.84	41.23±7.13	0.0119	0.1776
LPC(18:1)	231.84±63.24	268.76±77.1	0.0028	**0.0972**
LPC(18:2)	394.75±128.23	502.39±181.49	<0.0001	**0.0179**
LPC(20:3)	37.21±6.83	41.1±8.13	0.0003	**0.0269**
LPC(20:4)	63.03±14.89	66.84±15.93	0.0246	0.2282
PC(16:0e/18:1(9Z))	92±29.94	105.2±28.8	0.0368	0.2391
PC(30:0)	56.88±16.51	65.36±23.83	0.0165	0.1981
PC(32:0)	175.51±39.91	200.61±38.49	0.0083	0.1486
PC(32:1)	238.88±90.72	288.76±113.49	0.0231	0.2282
PC(32:2)	61.56±20.85	72.75±21.5	0.0027	**0.0972**
PC(34:2)	3759.47±717.83	4257.93±741.34	0.0065	**0.0972**
PC(36:2)	2940.24±630.99	3360.9±623.41	0.0125	0.1776
PC(36:3)	1753.26±669.47	2152.76±569.66	0.0349	0.2391
PC(36:4	404.62±165.27	504.24±198.32	0.0038	**0.0972**
PC(38:2)	70.5±22.97	81.8±22.28	0.0244	0.2282
PC(38:4)	36.07±10.37	40.94±10.62	0.0374	0.2391
PC(40:6)	56.74±20.41	64.26±17.7	0.0357	0.2391
PC(O-32:0)	49.78±11.18	55.84±11.43	0.0078	0.1486
PC(O-32:1)	37.88±7.19	40.82±7.11	0.0353	0.2391
PC(O-34:2)	85.95±27.42	98.13±30.17	0.0255	0.2282
PC(O-34:3)	111.62±33.27	132.51±36.51	0.0036	**0.0972**
PC(O-36:2)	51.25±15.7	57.48±14.77	0.0166	0.1981
PC(O-36:3)	42.86±11.11	47.38±11.72	0.0277	0.2361
PC(O-38:6)	74.25±23.67	84.11±22.81	0.0455	0.2627
SM(d18:0/16:0)	829.78±212.45	933.75±194.41	0.0129	0.1776
SM(d18:1/24:0)	6167.04±2537.94	7281.12±2418.58	0.0029	**0.0972**
SM(d41:1)	3540.52±1381.32	3889.15±1148.96	0.0419	0.25
SM(d42:2)	15796±5875.87	18218.69±5133.36	0.0339	0.2391
TG(56:7)	103.59±97.25	76.17±46.64	0.0198	0.2215

Abbreviations: CE, cholesterol ester; LPC, lysophosphatidylcholine; PC, phosphatidylcholine; PD, psychotic disorder; SM, sphingomyelin; TG, triacylglycerol.

Values are expressed as standard units±s.d. (standard deviation). *P*-values are adjusted for gender and BMI. FDR-adjusted *P*-values are adjusted for multiple comparisons using the Benjamini–Hochberg procedure. Values in bold are lipids that remain significant (*P*<0.1) folllowing correction for multiple comparisons.

**Table 3:** Description of lipid clusters obtained from lipidomics platform in the age 11 cohort and evaluated across the study groups

*Cluster name*	*Cluster size*	*Cluster description*	P-*value (controls* vs *cases)*
LC1	12	TGs	0.255
LC2	44	TGs	0.966
LC3	8	LPCs	**0.025**
LC4	29	PCs and TGs	0.96
LC5	23	PCs and SM	0.054
LC6	40	PCs and CEs	**0.036**
LC7	20	PCs	**0.006**

Abbreviations: CEs, cholesterol esters; LC, lipid cluster; LPCs, lysophosphatidylcholines; PCs, phosphatidylcholines; SM, sphingomyelins; TGs, triacylclycerols. Values in bold are lipid clusters (LC) that are significant between controls vs cases (*P*<0.05).

**Table 4:** Differential plasma lipids between control and PD groups at age 18

*Lipid name*	*Control (*n=*117)*	*Cases (*n=*36)*	P-*value*	*FDR-adjusted*
LPC(16:0)	848.58±191.96	771.44±227.83	0.0402	0.4042
LPC(16:1)	37.75±6.43	35.03±5.67	0.0319	0.3609
LPC(22:6)	28.63±4.66	25.7±4.46	0.0255	0.4196
PC(31:0)	26.39±7.66	22.9±7.38	0.0136	0.4103
PC(32:1)	281.87±137.89	249.65±106.1	0.0311	0.3752
PC(33:1)	62.63±18.43	54.06±13.57	0.0037	0.2232
PC(35:1)	75.59±21.09	68.31±13.57	0.0405	0.3858
PC(36:2)	67.41±21.4S	62.37±16.45	0.0468	0.385
PC(36:4)	2641.06±637.15	2656.62±708.18	0.0369	0.3929
PC(37:4)	67.93±26.92	59.57±28.94	0.0456	0.4127
PC(38:6)	114.34 ±42.33	109.33±32.87	0.0122	0.4416
PC(40:6)	283.19±122.05	255.9±115.8	0.031	0.4008
PC(40:7)	76±27.51	68.99±23.89	0.0147	0.3801
PC(O-38:4)	100.79±36.03	85.19±33.38	0.0213	0.4283
PC(O-40:6)	34.75±6.81	32.08±6.39	0.0169	0.3823
PC(O-42:3)	29.63±6.56	26.94±7.07	0.047	0.3699
PE(P-18:0/22:6)	35.6±8.05	32.61±7.62	0.0466	0.4016
SM(d18:0/16:0)	890.9±207.12	850.9±189.57	0.0118	0.534
SM(d32:1)	1901.22±547.7	1783.34± 498.81	0.0259	0.3907
SM(d33:1)	1227.82±311.1	1118.09±297.23	0.003	0.2715
TG(49:3)	33.78±1.18	33.31±1.53	0.0242	0.438
TG(51:2)	26.14±3.26	24.06±5.41	0.0024	0.4344
TG(56:7)	106.1±58.84	95.83±76.34	0.0288	0.401

Abbreviations: CE, cholesterol ester; LPC, lysophosphatidylcholine; PC, phosphatidylcholine; PD, psychotic disorder; SM, sphingomyelin; TG, triacylglycerol.

Values are expressed as standard units±s.d. (standard deviation). *P*-values are adjusted for gender and BMI. FDR-adjusted *P*-values are adjusted for multiple comparisons using the Benjamini–Hochberg procedure.

**Table 5:** Differential plasma metabolites between control and PD groups at age 11

*Metabolite name*	*Control (*n=*67)*	*Cases (*n=*38)*	P-*value*	*FDR-adjusted*
1-monopalmitin	448.89±162	368.91±123.4	0.0128	0.544
2,4-Dihydroxybutanoic acid	0.09±0.03	0.07±0.02	0.014	0.544
d-(−)-ribofuranose	98.34±169.78	52.87±40.02	0.0429	0.7
Ethanolamine	250.51±88.88	214.15±46.75	0.0215	0.544
Ribitol	175.9±41.79	155.67±31.77	0.0202	0.544
Hydroxylamine	88.11±38.61	66.9±39.02	0.0043	0.544
Sugar derivative	805.27±247.15	961.92±337.63	0.0274	0.591
Sugar derivative	89.91±74.69	141.94±85.59	0.0216	0.544

Abbreviations: BMI, body mass index; FDR, false discovery rate; PD, psychotic disorder. Values are expressed as standard units±s.d. (standard deviation). *P*-values are adjusted for gender and BMI. FDR-adjusted *P*-values are adjusted for multiple comparisons using the Benjamini–Hochberg procedure.

**Table 6:** Differential plasma metabolites between control and PD groups at age 18

*Metabolite name*	*Control (*n=*117)*	*Cases (*n=*36)*	P-*value*	*FDR-adjusted*
1-Monopalmitin	312.8±129.67	254.3±112.61	0.012	0.323
2-Hydroxybutyric acid	577.68±232.1	489.04±213.93	0.0161	0.323
3-Hydroxybutyric acid	36.64±37.35	25.76±23.78	0.0464	0.369
Cholesterol	359.7±82.18	335.36±75.08	0.0165	0.323
Citric acid	7.22±2.98	5.85±2.36	0.0329	0.346
Glycine	11.52±3.19	12.07±3.79	0.0374	0.346
Leucine	13.13±3.34	11.23±2.72	0.412	0.346
l-tryptophan	14.95±7.76	12.12±8.77	0.0411	0.346
Oleic acid	88.13±30.34	79.9±25.78	0.0344	0.346
Palmitic acid	105.31±23.19	97.2±16.08	0.0171	0.323
Scyllo-inositol	79.11±102.3	51.76±40.75	0.0215	0.325
Stearic acid	35.94±8.4	32.93±6.74	0.0275	0.346
Sugar derivative	1666.31±517.3	1474.28±530.76	0.0288	0.346
A144004	416±242.81	314.94±301.41	0.0125	0.323
Sugar derivative	1253.88±362.07	1076.74±395.7	0.0137	0.323
Threonic acid	1457.44±491.45	1294.07±599.63	0.02	0.325
Sugar derivative	916.23±283.49	840.44±324.57	0.0155	0.323
Tocopherol, α	453.34±307.71	353.09±133.91	0.0151	0.323
Levoglucosan	31.55±52.17	36.39±51.9	0.0356	0.346

Abbreviations: BMI, body mass index; FDR, false discovery rate; PD, psychotic disorder. Values are expressed as standard units±s.d. (standard deviation). *P*-values are adjusted for gender and BMI. FDR-adjusted *P*-values are adjusted for multiple comparisons using the Benjamini–Hochberg procedure.

**Table 7:** TCA cycle metabolite differences between study groups at age 18

*Metabolite*	*Controls (*n=*36) (mg l^−1^)*	*Cases (*n=*36) (mg l^−1^)*	P-*value*
Citric acid	11.68±5.86	9.43±4.84	0.07
Succinic acid	0.57±0.24	0.46±0.23	0.04
Fumaric acid	0.72±0.08	0.77±0.48	0.69
a-ketoglutaric acid	1.06±0.65	0.87±0.68	0.24
Malic acid	0.47±0.47	0.52±0.47	0.36

Abbreviations: BMI, body mass index; TCA, tricarboxylic acid. *P*-values are adjusted for gender and BMI.
