# Supplementary material for: Identification of a plasma signature of psychotic disorder in children and adolescents from the Avon Longitudinal Study of Parents and Children (ALSPAC) cohort
Source: Transl Psychiatry. 2017 Sep 26;7(9):e1240–. doi: 10.1038/tp.2017.211 (PMC5639252; doi:10.1038/tp.2017.211)
Supplement: Supplementary Tables [file tp2017211x1.docx]

**Supplementary Table 1**: Description of lipid clusters obtained from lipidomics platform in the Age 18 cohort and evaluated across the study groups

| Cluster name | Cluster size | Cluster description | P-value (controls vs cases) |
| --- | --- | --- | --- |
| LC1 | 31 | TGs | 0.275 |
| LC2 | 22 | TGs | 0.588 |
| LC3 | 25 | TGs | 0.162 |
| LC4 | 11 | LPCs | **0.017** |
| LC5 | 27 | PCs | 0.071 |
| LC6 | 27 | PCs | 0.236 |
| LC7 | 36 | CEs & SM | 0.629 |

LC: lipid cluster; TGs: triacylclycerols; LPCs: lysophosphatidylcholines; PCs: phosphatidylcholines; SM: sphingomyelins; CEs: cholesterol esters

**Supplementary Table 2**: Description of metabolite clusters obtained from metabolomics platform in the Age 11 cohort and evaluated across the study groups

| Cluster name | Cluster size | Cluster description | P-value (controls vs cases) |
| --- | --- | --- | --- |
| CL1 | 22 | Mainly unknowns | 0.734 |
| CL2 | 30 | Amino acids and unknowns | 0.797 |
| CL3 | 28 | Fatty acids | 0.773 |
| CL4 | 8 | Unknowns | 0.384 |
| CL5 | 27 | Mainly unknowns | 0.961 |
| CL6 | 36 | Organic acids, amino acids & unknowns | 0.447 |

CL: metabolite cluster

**Supplementary Table 3**: Description of metabolite clusters obtained from metabolomics platform in the Age 18 cohort and evaluated across the study groups

| Cluster name | Cluster size | Cluster description | P-value |
| --- | --- | --- | --- |
| CL1 | 22 | Fatty acids, organic acids & unknowns | 0.034 |
| CL2 | 37 | Mainly unknowns & amino acids | 0.711 |
| CL3 | 8 | Mainly unknowns | 0.442 |
| CL4 | 24 | Amino acids | 0.931 |
| CL5 | 12 | Mainly unknowns | 0.186 |
| CL6 | 20 | Mainly unknowns | 0.176 |

CL: metabolite cluster

**Supplementary Table 4**: Overview of the ALSPAC study subset used for the targeted metabolomic analysis

|  |  | Controls Age 11 (n=38) | Cases Age 11 (n=38) |  | Controls Age 18 (n=36) | Cases Age 18 (n=36) |
| --- | --- | --- | --- | --- | --- | --- |
| BMI ± SD |  | 18.07 ± 2.69 | 18.2 ± 3.36 |  | 23.37 ± 3.17 | 23.12 ± 3.74 |
| Male: Female |  | 10 M: 28 F | 8 M: 30 F |  | 8 M: 28 F | 8 M: 28 F |

BMI: body mass index; SD: standard deviation
