# Supplementary material for: Identification of a plasma signature of psychotic disorder in children and adolescents from the Avon Longitudinal Study of Parents and Children (ALSPAC) cohort
Source: Transl Psychiatry. 2017 Sep 26;7(9):e1240–. doi: 10.1038/tp.2017.211 (PMC5639252; doi:10.1038/tp.2017.211)
Supplement: Supplementary Figures [file tp2017211x2.pdf]

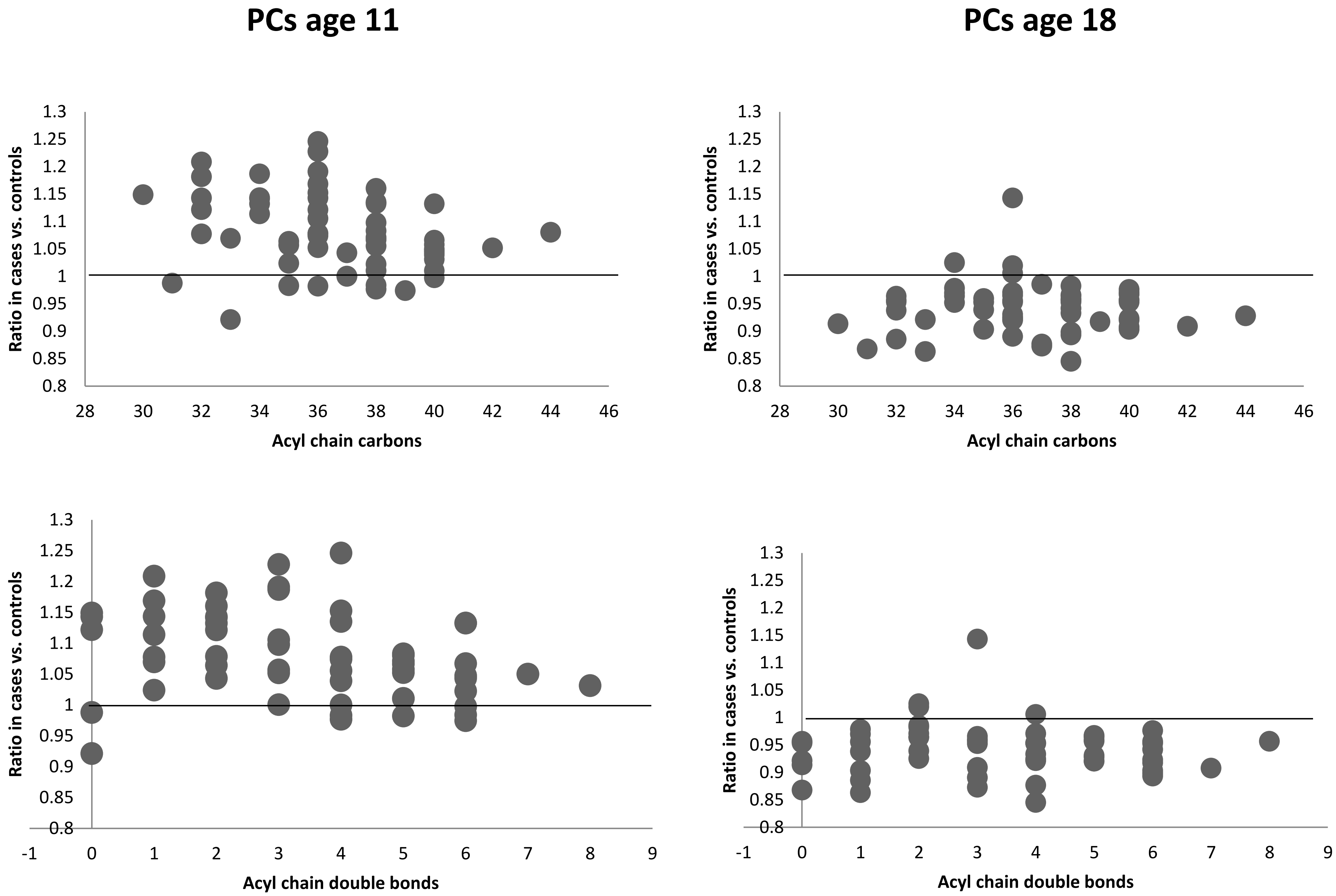

PCs: Phosphatidylcholines

Supplementary Figure 1: Relationship between PD and acyl chain content for PCs.

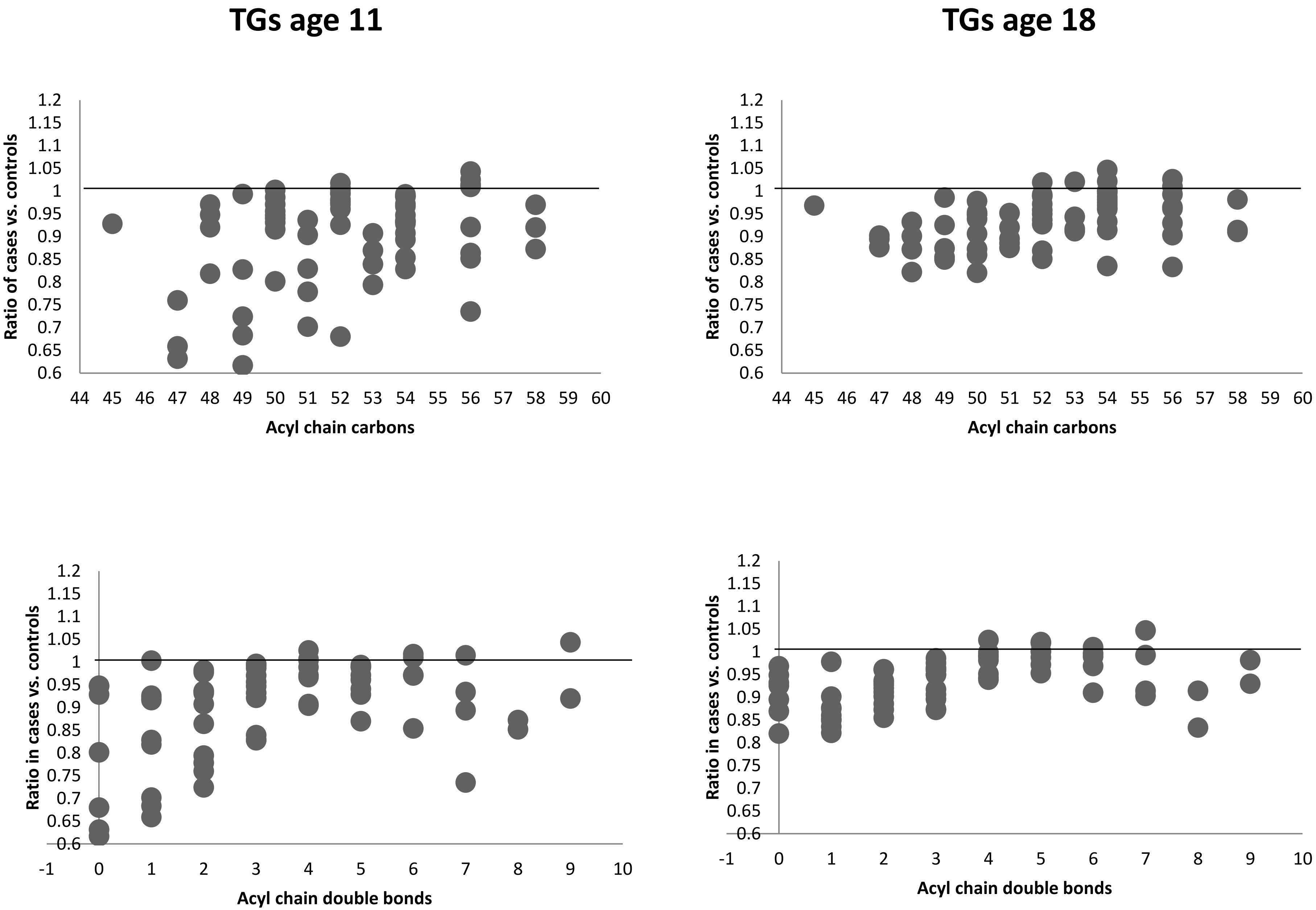

TAGs: Triacylglycerides

Supplementary Figure 2: Relationship between PD and acyl chain content for TAGs.

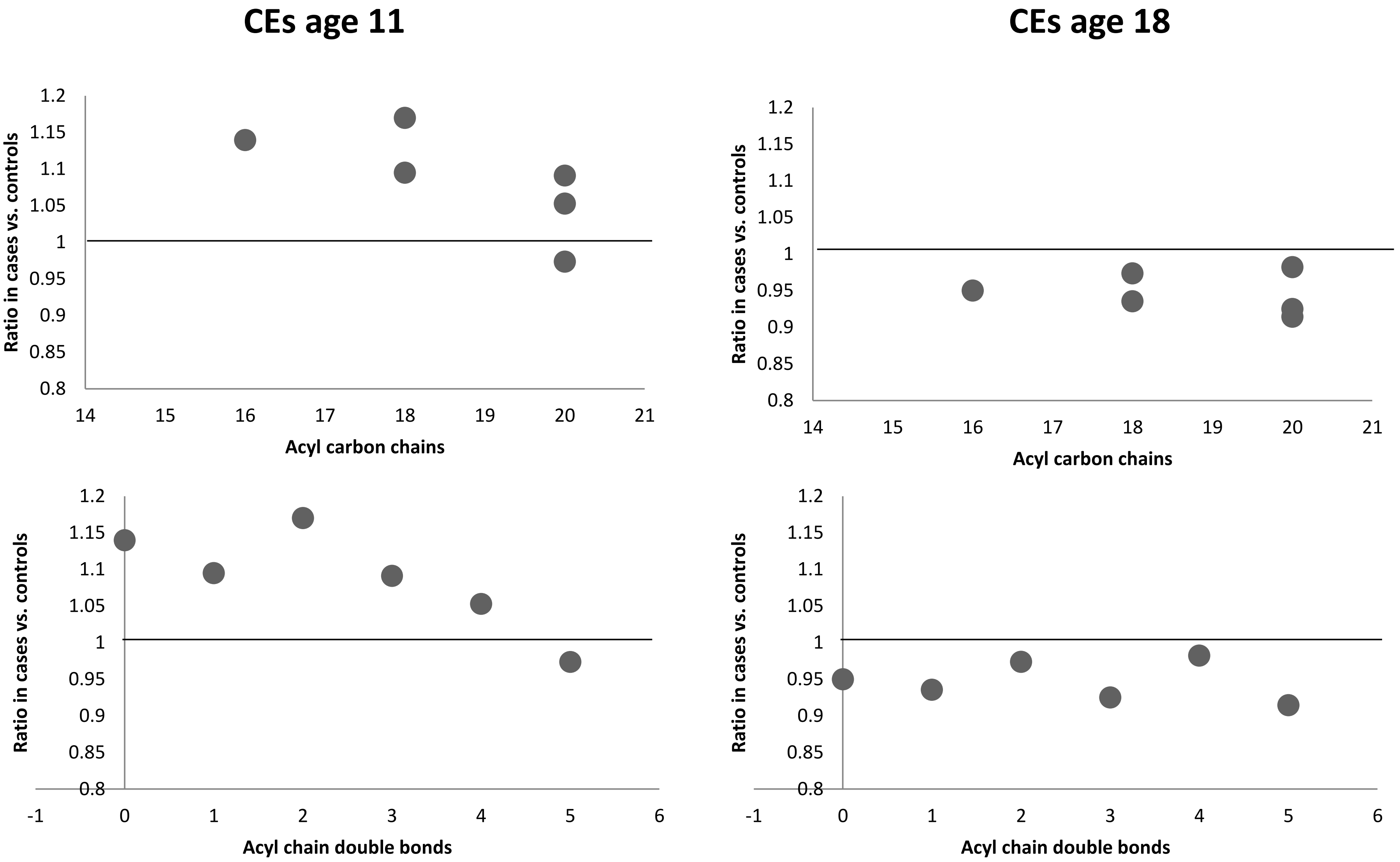

CEs: Cholesterol esters

Supplementary Figure 3: Relationship between PD and acyl chain content for CEs.
